# Complement-Mediated Virus Infectivity Neutralisation by HLA Antibodies Is Associated with Sterilising Immunity to SIV Challenge in the Macaque Model for HIV/AIDS

**DOI:** 10.1371/journal.pone.0088735

**Published:** 2014-02-14

**Authors:** Mark Page, Ruby Quartey-Papafio, Mark Robinson, Mark Hassall, Martin Cranage, James Stott, Neil Almond

**Affiliations:** 1 Division of Virology, National Institute of Biological Standards and Control, South Mimms, Potters Bar, Herts, United Kingdom; 2 Centre for Infection & Immunity, Division of Clinical Sciences, St George’s, University of London, London, United Kingdom; Imperial College London, United Kingdom

## Abstract

Sterilising immunity is a desired outcome for vaccination against human immunodeficiency virus (HIV) and has been observed in the macaque model using inactivated simian immunodeficiency virus (SIV). This protection was attributed to antibodies specific for cell proteins including human leucocyte antigens (HLA) class I and II incorporated into virions during vaccine and challenge virus preparation. We show here, using HLA bead arrays, that vaccinated macaques protected from virus challenge had higher serum antibody reactivity compared with non-protected animals. Moreover, reactivity was shown to be directed against HLA framework determinants. Previous studies failed to correlate serum antibody mediated virus neutralisation with protection and were confounded by cytotoxic effects. Using a virus entry assay based on TZM-bl cells we now report that, in the presence of complement, serum antibody titres that neutralise virus infectivity were higher in protected animals. We propose that complement-augmented virus neutralisation is a key factor in inducing sterilising immunity and may be difficult to achieve with HIV/SIV Env-based vaccines. Understanding how to overcome the apparent block of inactivated SIV vaccines to elicit anti-envelope protein antibodies that effectively engage the complement system could enable novel anti-HIV antibody vaccines that induce potent, virolytic serological response to be developed.

## Introduction

The ability to induce virus-neutralising antibodies is considered a key property for an efficacious HIV/AIDS vaccine [1], [2]. This will be particularly critical for protection against infection with HIV as, once the virus gains access to the lymphoid system; it spreads rapidly and establishes pockets of latency through the integration of proviral DNA. Thus, unlike for most existing vaccines, for HIV it may be necessary to establish sterilising immunity. However, the properties of antibodies induced by vaccination that can confer potent protection remain poorly defined. Anti-envelope antibodies appear to neutralise primarily through the blocking of interaction of the viral envelope protein with its receptor CD4 [3].

In animal models, such antibodies have been demonstrated to protect against infection, but they require high titres or very high affinity to be effective, which can be difficult to attain in all vaccine recipients [4]–[8]. In clinical vaccine research, whilst anti envelope protein antibodies are currently perceived to be a desired outcome; most emphasis is being placed on characterising the specificity of antibodies that are able to bind a broadly divergent range of HIV-1 envelope proteins with high affinity [9], [10]. These antibodies have been derived from infected individuals, who remain unable to clear the virus. By contrast only limited effort is being focussed on characterising the functional properties of antibodies that have been demonstrated to protect solidly against virus challenge.

Other experimental AIDS vaccines have also been shown to mediate protection in an antibody dependent manner. Early studies in simian models used fixed inactivated virus vaccines, where solid protection against wild-type virus challenge was reported by a number of groups [11]–[16]. This vaccine-mediated protection was shown to be transferable with immune serum alone [14]. Critically, however, it became apparent that the key vaccine components were not viral-encoded antigens, but host cell proteins that were present in the vaccine preparations derived from the human cellular substrates used [17]–[20]. Moreover, it was demonstrated that immunization with HLA class I [21] or HLA class II [22] protected a proportion of macaques against challenge with human cell-grown SIV. However, there were limited analyses of the mechanism of virus neutralisation, since the antibodies were induced by xeno-immunisation and were unable to protect macaques against virus propagated on simian cells [23]–[25]. Nonetheless, these results highlight the potential of anti-virion antibodies to mediate protection against virus infection *in vivo*. By understanding more fully the mechanisms by which antibodies directed against host cell proteins on the virion can confer sterilising protection, it may be possible to design potent, novel, clinically acceptable anti-HIV vaccines.

For such a mechanism to operate in humans, allo-immunisation must be effective against HIV. Some studies suggest that this is possible where alloimmunisation of women with their partner’s cells induced HIV resistance in vitro [26] and the sera neutralised HIV when grown in their partner’s white blood cells [27]. Further, sera from polytransfused patients generating allo-antibodies (including HLA antibodies) were able to neutralise HIV grown in cells expressing corresponding HLA [28]. Our own studies have also shown that immunisation with a fixed inactivated HIV vaccine produces anti HLA antibody responses that are concordant with the host cell line used to produce the vaccine [29].

The failure of current vaccine strategies to elicit antibodies that protect with the potency of those induced by fixed inactivated viruses, led us to apply techniques that were unavailable at the time of the original vaccine studies, to analyse the properties of protective sera elicited by fixed inactivated SIV vaccines further. Here we report that protection with fixed inactivated vaccines correlates with the presence of antibodies directed against framework determinants on MHC Class I and II molecules and also with antibodies that neutralise virus through host cell components in the presence of complement. By contrast, neutralisation by anti SIV envelope protein antibodies generated by the same vaccines did not demonstrate any capacity to harness complement. These data identify a strategy to develop more potent vaccines through their ability to elicit anti-HIV antibody responses that are able to effectively engage complement.

## Materials and Methods

### Animals

Juvenile, purpose bred, cynomolgus macaques (Macaca fascicularis) were used in strict accordance with UK Home Office guidelines. Work at NIBSC is governed by the Animals (Scientific Procedures) Act 1986 which complies with the EC Directive 86/609 and performed under licence PPL 80/1952 granted only after review of all the procedures in the licence by the NIBSC Animal Welfare and Ethical Review Body. All study macaques were purpose bred in the UK and group-housed for the entire duration of the study. Macaques are fed primarily with a formulated pelleted diet, sourced from a specialist UK diet manufacturer. This diet is intended to be nutritionally complete and balanced for Old World Primates, but is also supplemented with additional food items, including a range of fresh fruit and vegetables to give variety. Food is presented in measured amounts at varying time points during the day, using a variety of presentation methods to encourage foraging behaviours. Food intake is monitored and animals are weighed regularly to check for instances of over -or under-consumption.

All animals were sedated with ketamine prior to bleeding or virus inoculation by venepuncture. Frequent checks were made by staff and any unexpected change in behaviour by individuals on study followed up, including seeking of veterinary advice where necessary. Regular blood samples were obtained to assess haematological parameters in blood that might provide evidence of incipient disease and veterinary advice sought when persisting abnormalities detected. The study was terminated and all animals killed humanely by administering an overdose of pentobarbital (200 mg/ml) by intra-cardiac puncture. All efforts were made to minimise animal suffering.

### Vaccines

Formalin-inactivated SIVmac_251_32H was prepared as described previously [30]. The vaccine was formulated in RIBI as adjuvant and administered at 500 µg or 100 µg doses. This work was performed as part of a European wide study [31].

A fixed uninfected C8166 cell based vaccine was prepared as described previously [32]. Briefly 2×10^6^ cells were fixed in 0.15% glutaraldehyde and 0.2% β-propiolactone and formulated with 100 µg of Quil A or GMDP as adjuvants. A fixed SIVmac_251_32H infected C8166 cell based vaccine was prepared and administered as described previously [16].

All vaccines were administered sub-cutaneously in the quadriceps muscles.

### Serology

Sera used in these studies were stored frozen at −20°C since the time of the vaccine studies carried out from 1990 and later.

### HLA Bead Array Assay

For analysis of the antibody specificity, a Lifematch Lifecodes class I and class II ID (Tepnel Life Sciences) kit consisting of beads coated with pools of either MHC class I or MHC class II glyco-proteins was used. Serum samples were incubated with the beads following manufacturer’s instructions and analysed on a Luminex® machine [29].

### Virus Infectivity Neutralisation Assay

Neutralising antibody activity was determined using TZM-bl cells and an adaptation of the method of Wei et al., (2002) [33]. The test serum which had been incubated at 56°C for 1 hour was serially diluted in 2 fold steps in DMEM containing 10% v/v foetal calf serum (FCS) in 96 well microtitre plates. This was performed in triplicate. Fifty µl of virus stock containing twice the fifty percent culture infectious dose (TCID_50_) for each virus (predetermined on TZM-bl cells) was added to all wells and the antibody/virus mixture incubated for 45 minutes. 20 µl of normal macaque serum which had not been heat treated was added as a source of autologous complement and the mixture incubated for a further 15 minutes. Replicate plates were set up with heat-treated (56°C for 1 hour) normal macaque serum to control for complement activity. The indicator cell line TZM-bl which expresses β-galactosidase and luciferase on infection with HIV or SIV was added to the wells (10,000 cells per well) and the plate incubated at 37°C for 48 hours.

Following incubation, the supernatant was removed by aspiration and the cells lysed by the addition of lysis buffer containing 5% NP40. The presence of β galactosidase in the cell lysate was determined using a Novagen β-red β-galactosidase kit (Novagen Inc.) and following the manufacturer’s instructions. The colour development was terminated using the kit stop solution and the absorbance at 590 nm determined using a Fluostar Omega microplate spectrophotometer (BMG Labtech, Ortenburg, Germany).

Anti-class I and II monoclonal antibodies were included in some assays to confirm HLA specific neutralization. W6/32(class I) and HL-39 (class II) mabs were used and derived from AbD Serotec, Oxford, UK.

### Viruses and Cells

The following viruses were used in neutralisation assays; SHIV_W61D_ [34] SIVsme660 [35]. These viruses were propagated either on the human CD4^+^ human lymphoblastoid cell line C8166; C8166 cells are a subclone of CR63/CR-4 cells derived by cell hybridization, and transformed *in vitro* by co-cultivation with HTLV-I producing cells [36]. Alternatively, viruses were propagated on HSC-F cells, a cynomolgus monkey CD4^+^ T-cell line from a foetal splenocyte that was immortalized by infection with *Herpesvirus saimiri* subtype C [37].

### Virus Detection *ex vivo*

Virus isolation from peripheral blood mononuclear cells (PBMC) was determined by co-culture with C8166 cells [38]; the presence of replicating virus was confirmed by visual identification of syncitia and SIVGagp24 antigen capture assay [39]. The presence of SIV in DNA isolated from whole blood was determined using SIV*gag* DNA PCR assay [39].

## Experimental Outline

**Figure 1:**
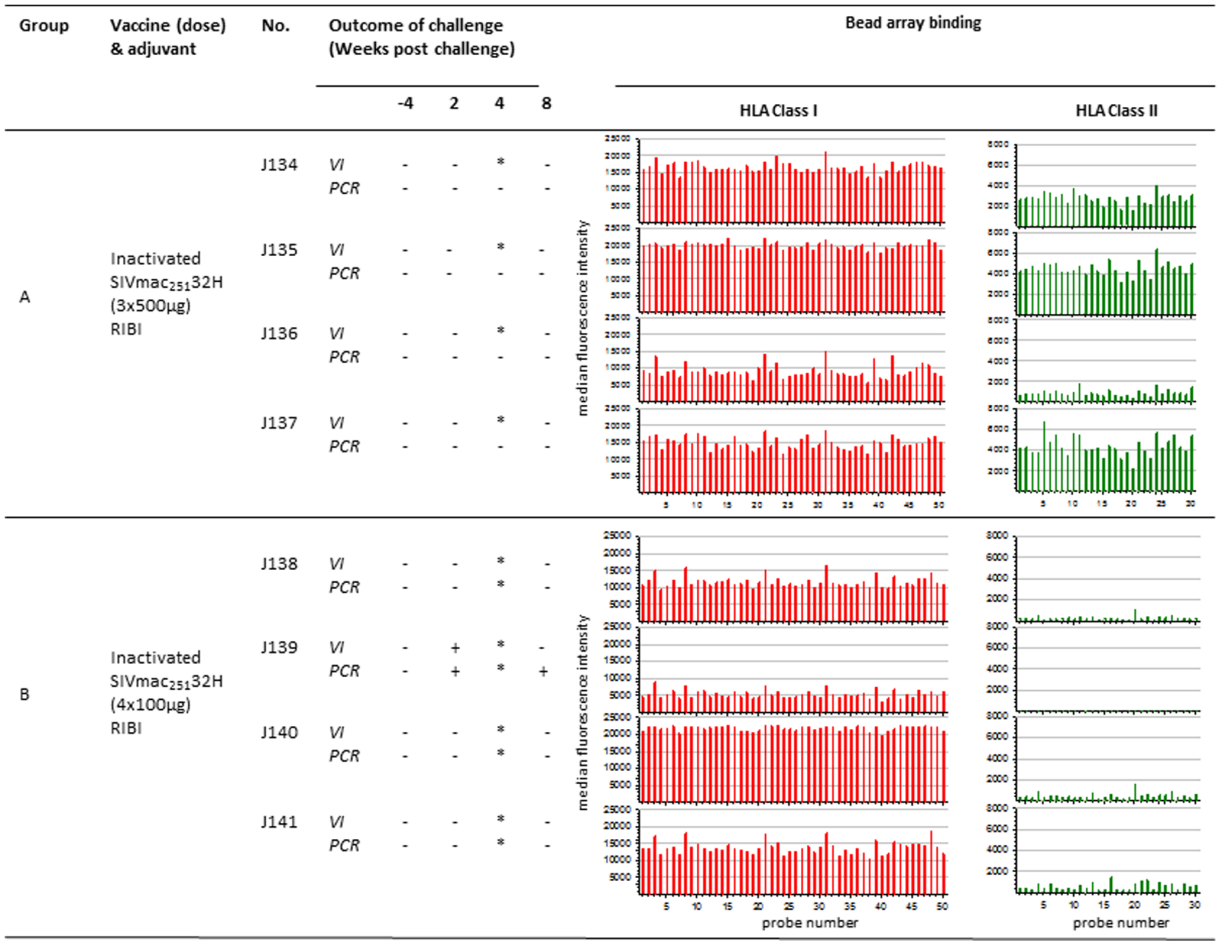
Association between anti-HLA reactivity following vaccination with formalin-inactivated SIV and outcome of challenge. Virus infection status was determined by PCR for SIV*gag* proviral DNA and virus isolation (VI) from PBMC of macaques challenged with SIVmac_251_32H (a) following vaccination with inactivated SIV with either 3×500 µg doses (group A) or 4×100 µg doses (group B). Bar charts show the associated median fluorescence intensity (y axis) of serum samples for each macaque tested against HLA class I (red bars) and class II (green bars) bead sets (x axis). Background binding of pre-vaccination sera is shown as light red and light green for HLA class I and class II respectively. * indicates not tested, probe number refers HLA allele-specific bead sets.

**Figure 2:**
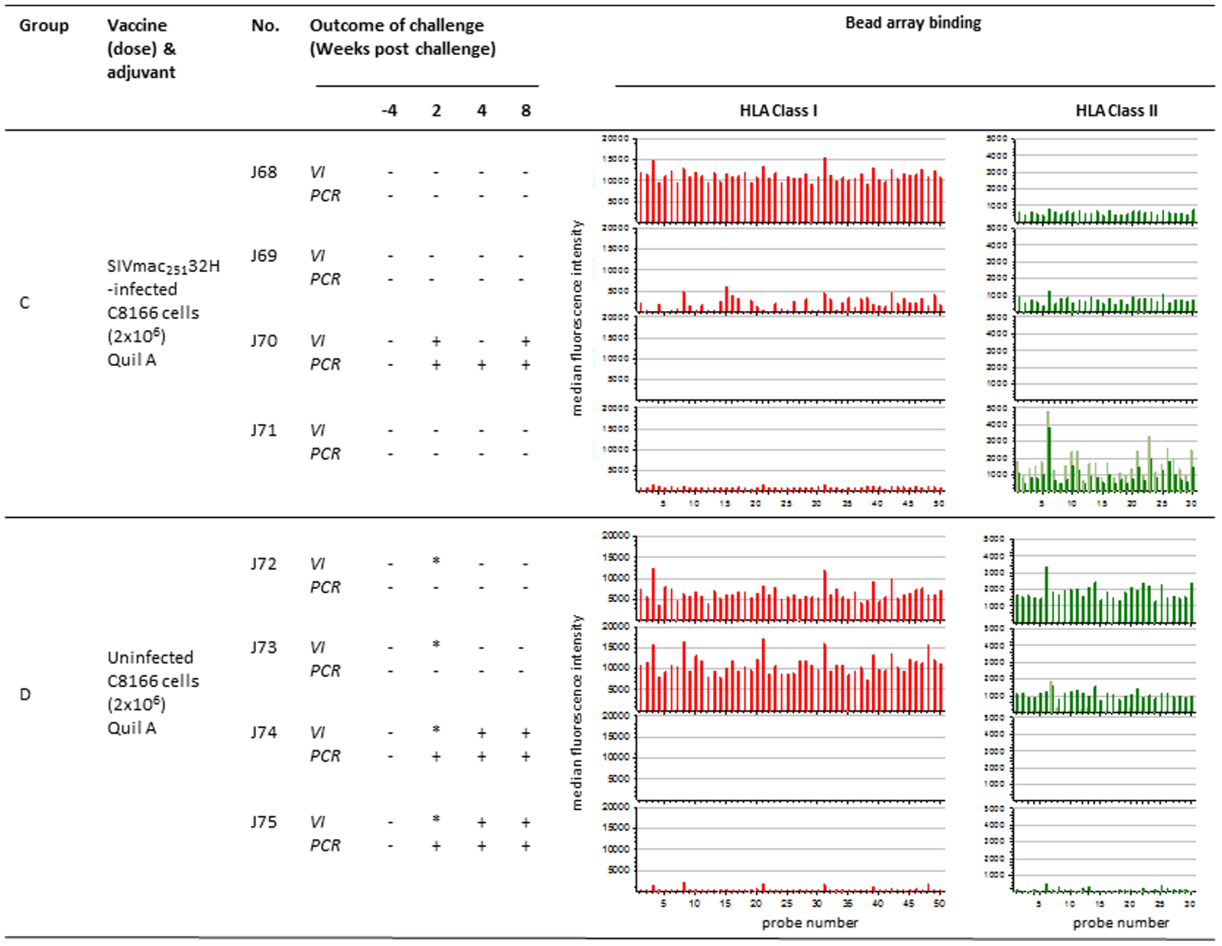
Association between anti-HLA reactivity following vaccination with fixed-inactivated SIV infected and uninfected C8166 cells and outcome of challenge. Virus infection status was determined by PCR for SIV*gag* proviral DNA and virus isolation (VI) from PBMC of macaques challenged with SIVmac_251_32H (a) following vaccination with 2 doses of SIV infected C8166 cells (group C) or with uninfected C8166 cells (group D). Bar charts show the associated median fluorescence intensity (y axis) of serum samples for each macaque tested against HLA class I (red bars) and class II (green bars) bead sets (x axis). Background binding of pre-vaccination sera is shown as light red and light green for HLA class I and class II respectively. * indicates not tested, probe number refers HLA allele-specific bead sets.

**Figure 3:**
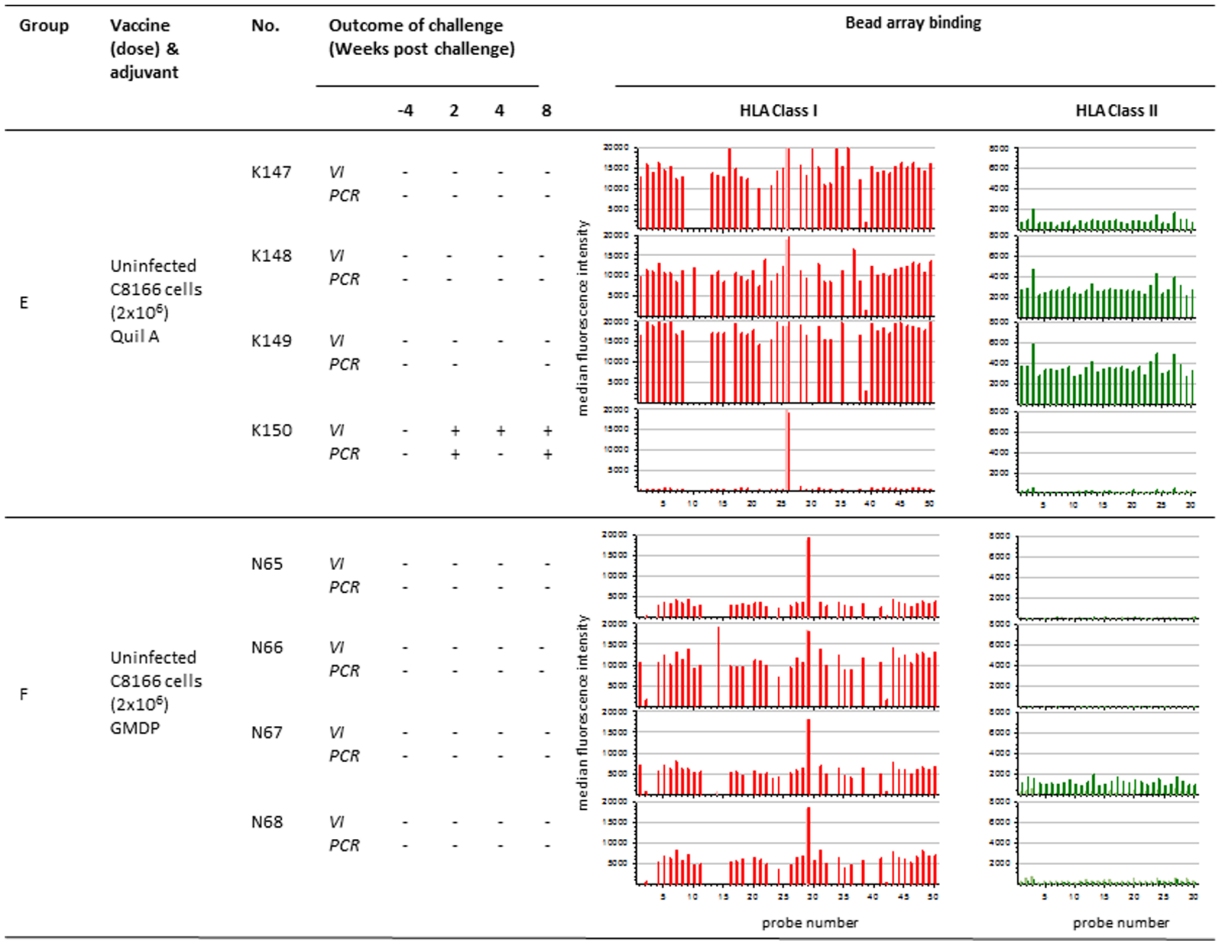
Association between anti-HLA reactivity following vaccination with uninfected C8166 cells using different adjuvants and outcome of challenge. Virus infection status was determined by PCR for SIV*gag* proviral DNA and virus isolation (VI) from PBMC of macaques challenged with SIVmac_251_32H (a) following vaccination with 4 doses of uninfected C8166 cells with Quil A adjuvant (group E) or with GMDP adjuvant (group F) (a) following vaccination with 2 doses of SIV infected C8166 cells (group C) or with uninfected C8166 cells (group D). Bar charts show the associated median fluorescence intensity (y axis) of serum samples for each macaque tested against HLA class I (red bars) and class II (green bars) bead sets (x axis). Background binding of pre-vaccination sera is shown as light red and light green for HLA class I and class II respectively. * indicates not tested, probe number refers HLA allele-specific bead sets.

The vaccine studies using inactivated SIV or uninfected cell vaccines are summarised in Figures 1, 2, 3. Two groups of 4 macaques (Groups A and B) were administered high (500 µg) or low (100 µg) doses of inactivated SIVmac_251_32H formulated in RIBI adjuvant (Figure 1). Group A received 3 immunisations on weeks 0, 4 and 8. Group B received 4 immunisations on weeks 0, 4, 8 and 16. This was performed as part of a European multicentre SIV vaccine study which has been reported previously [31].

Another 4 separate study groups (C-F) of 4 macaques each were also undertaken. Group C were immunised on two occasions at weeks 0 and 4 with 2×10^6^ fixed, inactivated C8166 cells infected with SIVmac_251_32H (Figure 2). Group D were immunised as for group C except uninfected cells were used (Figure 2). Group E were immunised as for group C except 4 immunisations were administered instead of two at weeks 0, 4, 8 and 16 (Figure 3). Group F were immunised as for group D except that GMDP was used as adjuvant (Figure 3).

Macaques were challenged intravenously two weeks after the last dose of vaccine with 10MID_50_ of the 11/88 pool of SIVmac_251_32H [30]. Groups of 4 naïve macaques were challenged contemporaneously as controls. The outcome of challenge was monitored by collection of blood samples at weeks 2, 4, 8 and 12 weeks after challenge and looking for evidence of virus by co-culture with indicator cells and diagnostic SIV specific DNA PCR [39].

## Results

### Outcome of Challenge with SIVmac_251_32H

All animals were virus negative by both co-culture for virus growth and PCR for *gag* prior to challenge (Figures 1, 2, 3). Following intravenous challenge with SIVmac_251_32H, all naive challenge controls (n = 16) became infected, detectable by both re-isolation of virus from PBMC by co-culture and by SIV-specific DNA PCR at multiple timepoints. Following challenge with SIVmac_251_32H, virus was not detected at any time-point in the blood of any macaques in group A (J134–J137 (Figure 1). In group B, virus was not detected in the blood at any time of individuals J138, J140 and J141. However for macaque J139, virus was detected at weeks 2 and 8 (Figure 1). Following challenge of macaques in Group C (J68–J70), there was no evidence of SIV in J68, J70 and J71. Virus was detected in J69 at 2 weeks after challenge (Figure 2). Amongst the macaques vaccinated with uninfected C8166 cell based vaccines, there was no evidence of virus detected in the blood of J72 and J73 in group D (Figure 2), K147–K149 in group E and N56–N59 in group F (Figure 3). For the other macaques immunised with uninfected C8166 cells virus was detected in the blood at multiple times following challenge by both virus isolation and PCR methods. These animals were as follows: J74 and J75 (group D; Figure 2), K150 (group E; Figure 3).

### Immune Response after Vaccination with Inactivated SIV or SIV-infected C8166 Cells

#### Anti-HLA response

Analysis of the specificity of anti-HLA responses was performed using Luminex® technology and bead sets covering a range of Class I and Class II alleles.

The anti-HLA response was characterised by a broad reactivity to all bead sets of both HLA class I and class II (Figures 1, 2, 3). A dose response was also observed where the median fluorescence intensities were slightly higher for group A receiving 500 µg SIV dose than group B vaccinated with100µg dose (not shown). Although the individual responses were variable in intensity, the lowest responders were observed to be J139 (group B; Figure 1) and J70 (group C; Figure 2) for both class I and class II; these were the macaques that became infected when challenged with virus.

#### Virus infectivity neutralising antibody

Heat inactivated sera, collected on the day of virus challenge from macaques that had been vaccinated with fixed inactivated SIV (Groups A and B - J134–J141), were evaluated for the ability to neutralise SHIV_W61D_ propagated on C8166 cells. Neutralising activity, that could be titrated, was detectable against this virus in all of the sera collected on the day of challenge (Figure 1) whereas pre-immune sera showed no activity. When fresh macaque serum was included in neutralisation assays as a source of complement, the neutralising titres increased. However the titres in serum from animals that became infected after challenge (J139; group B and J70; group C) were readily distinguishable from their contemporaries (figure 1). This difference was significant (ρ = 0.034; Mann Whitney U-test). The addition of complement to the neutralisation assay also resolved differences in the titres detected in the different vaccine groups; serum from groups A and B (vaccinated with inactivated SIV) exhibited more potent neutralisation at low serum dilutions compared with group C that was vaccinated with SIV infected C8166 cells. HLA specific neutralising activity was confirmed with anti-class I and class II monoclonal antibodies W6/32 and HL-39 respectively (data not shown).

### Immune Response after Vaccination with Uninfected C8166 Cells

#### Anti-HLA response

**Figure 4:**
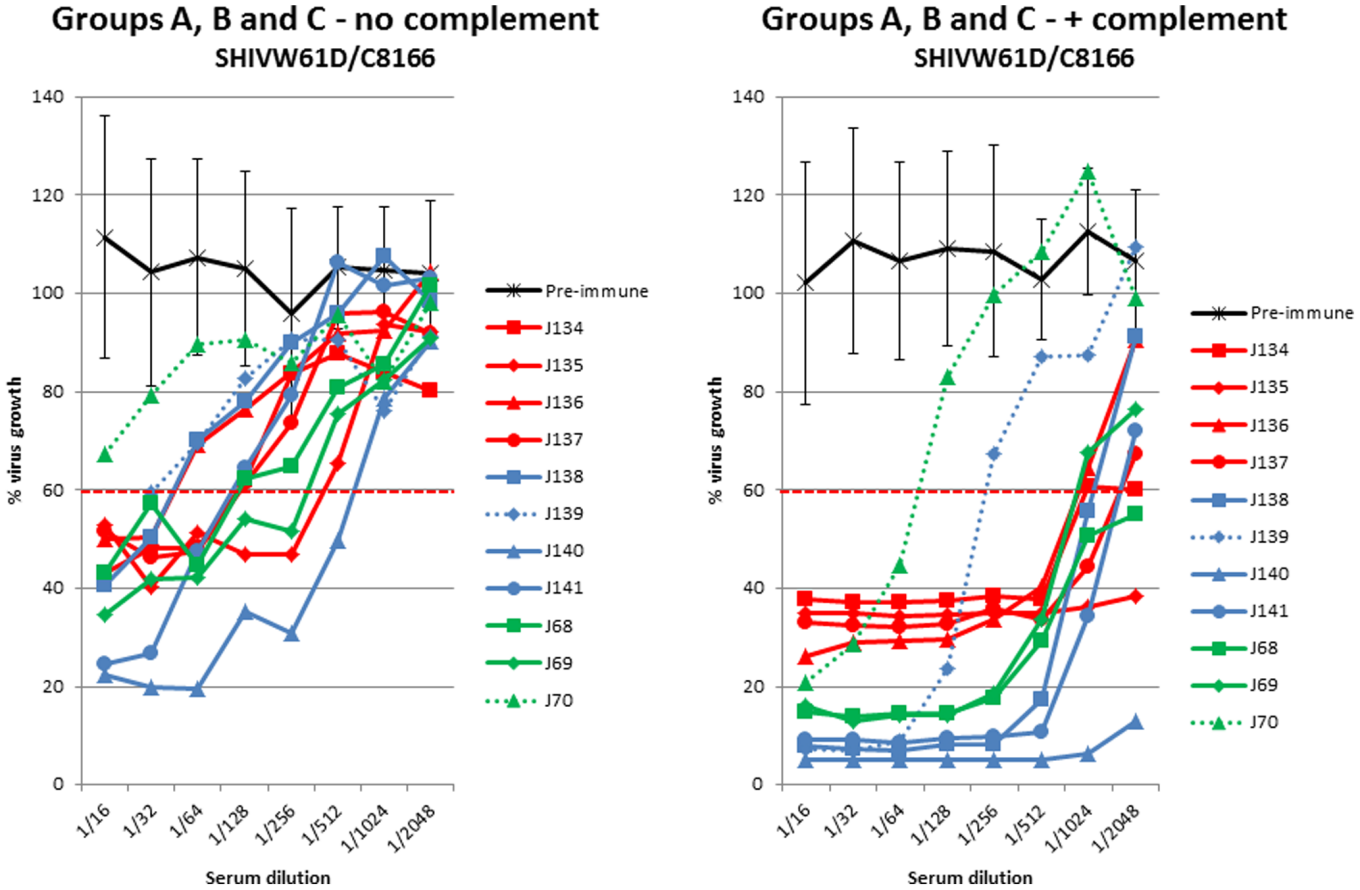
Virus infectivity neutralising activity following vaccination with inactivated SIV. Neutralising antibody activity against SHIV_W61D_ propagated on human C8166 cells in sera from macaques immunised with SIV-containing vaccines in the absence (left panel) or presence (right panel) of complement. Pre-immune sera (black line) are means of all animals with standard deviations. Group A immunised with 500 µg of inactivated SIV (red lines); group B immunised with 100 µg of inactivated SIV (blue lines) group C immunised with SIV-infected C8166 cells (green lines). Dashed lines indicate unprotected animals.

**Figure 5:**
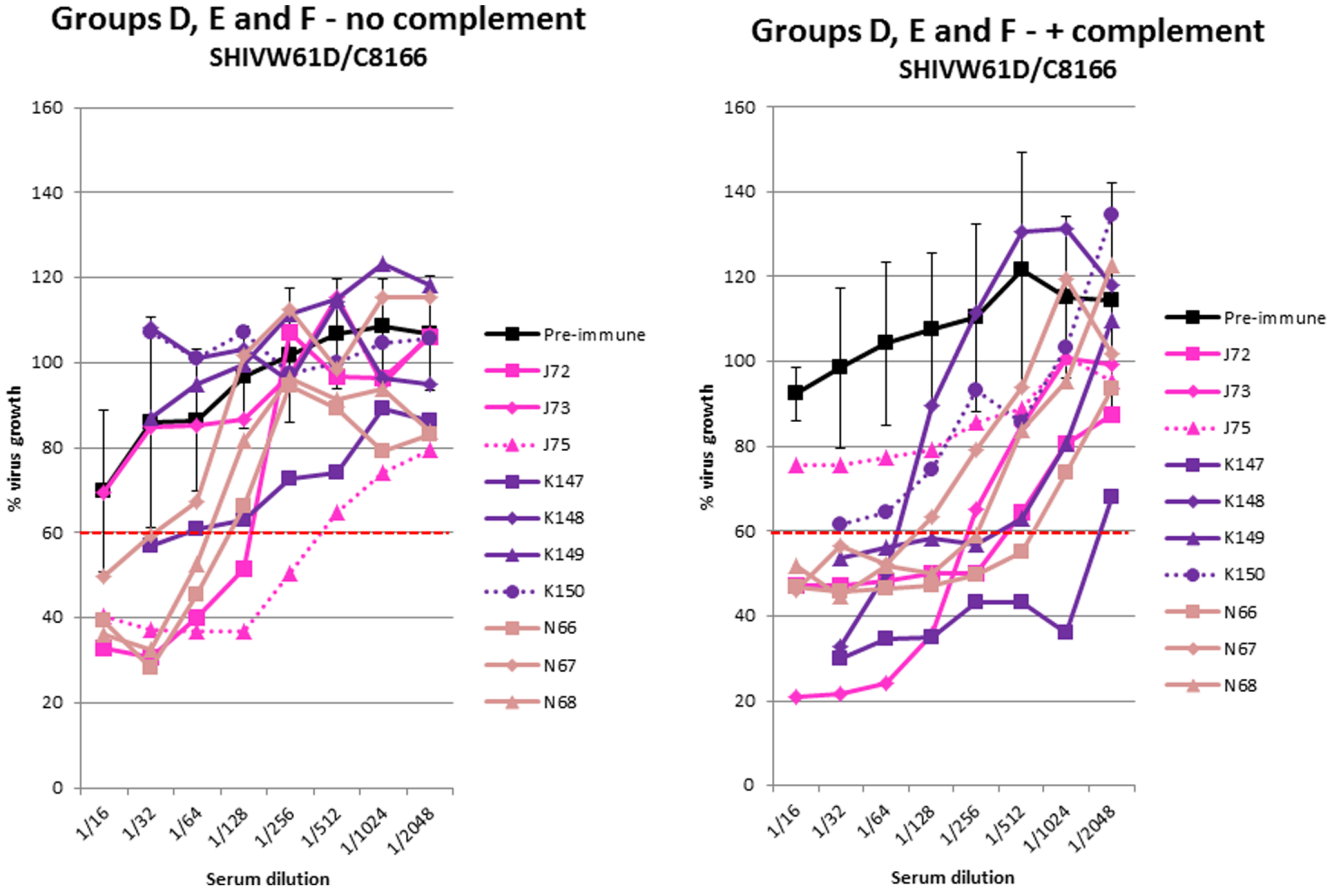
Virus infectivity neutralising activity following vaccination with uninfected cells. Neutralising antibody activity against SHIV_W61D_ propagated on human C8166 cells in sera from macaques immunised with uninfected C8166 cell vaccines in the absence (left panel) or presence (right panel) of complement. Pre-immune sera (black line) are means of all animals with standard deviations. Group D (pink lines); group E (purple lines) group F (pale brown lines). Dashed lines indicate unprotected animals.

**Figure 6:**
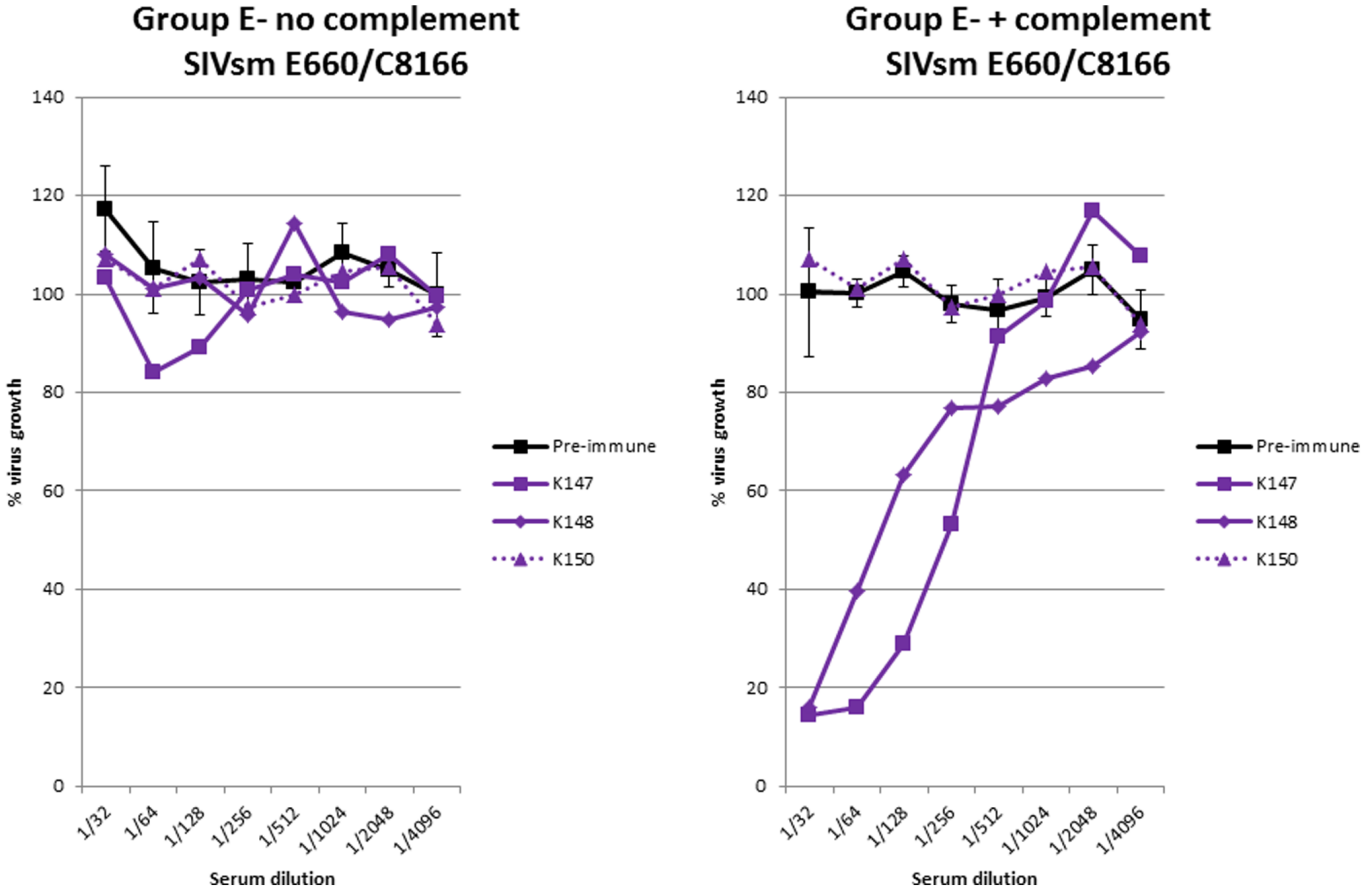
Infectivity neutralising activity against virus unrelated to vaccine. Neutralising antibody activity against SIVsmE660 propagated on human C8166 cells in sera from macaques immunised with uninfected C8166 cell vaccines (group E) in the absence (left panel) or presence (right panel) of complement. Pre-immune sera (black line) shows means of all animals with standard deviations. Dashed lines indicate unprotected animal.

Following vaccination uninfected C8166 cells, similar anti–HLA Class I and II responses were detected as those observed following immunisation with inactivated SIV (Figs. 4, 5, 6). Broad reactivity to all class I and II bead sets was observed. However, the vaccinated macaques which became patently infected after SIV challenge (J75, group D and K150, group E) had the lowest median fluorescence intensity within their vaccine groups.

#### Virus infectivity neutralising antibody

A similar profile of complement mediated neutralising activity was observed amongst macaques immunised with uninfected C8166 cells to those immunised with inactivated SIV vaccines (fig. 5). In the absence of complement, there was neutralising activity (<60% viral growth) in some animals of all the vaccine groups except animals K148, K149 and K150 (group E) and J73 (group D). With complement, neutralising activity at <60% viral growth was achieved in all sera except those from the unprotected animals (J75 and K150; serum for J74 was not available for analysis) (Fig. 5). This difference did not quite reach significance at the 5% level (ρ = 0.059; Mann Whitney U test).

Serum neutralising activity was tested against a different virus strain (SIVsmE660) that had been propagated on C8166 cells. No neutralising activity was detected in the sera of macaques from group D (K147, K148 and K150; figure 6), in the absence of complement. However, when complement was added, serum neutralising activity against this second virus was detectable in all sera except from macaque K150 that was not protected when challenged with SIVmac_251_32H. For this individual, sera collected after immunisation exhibited neutralising titres similar to pre-immune sera.

### Effect of Cell Substrate on Complement-mediated Virus Infectivity Neutralising Activity

**Figure 7:**
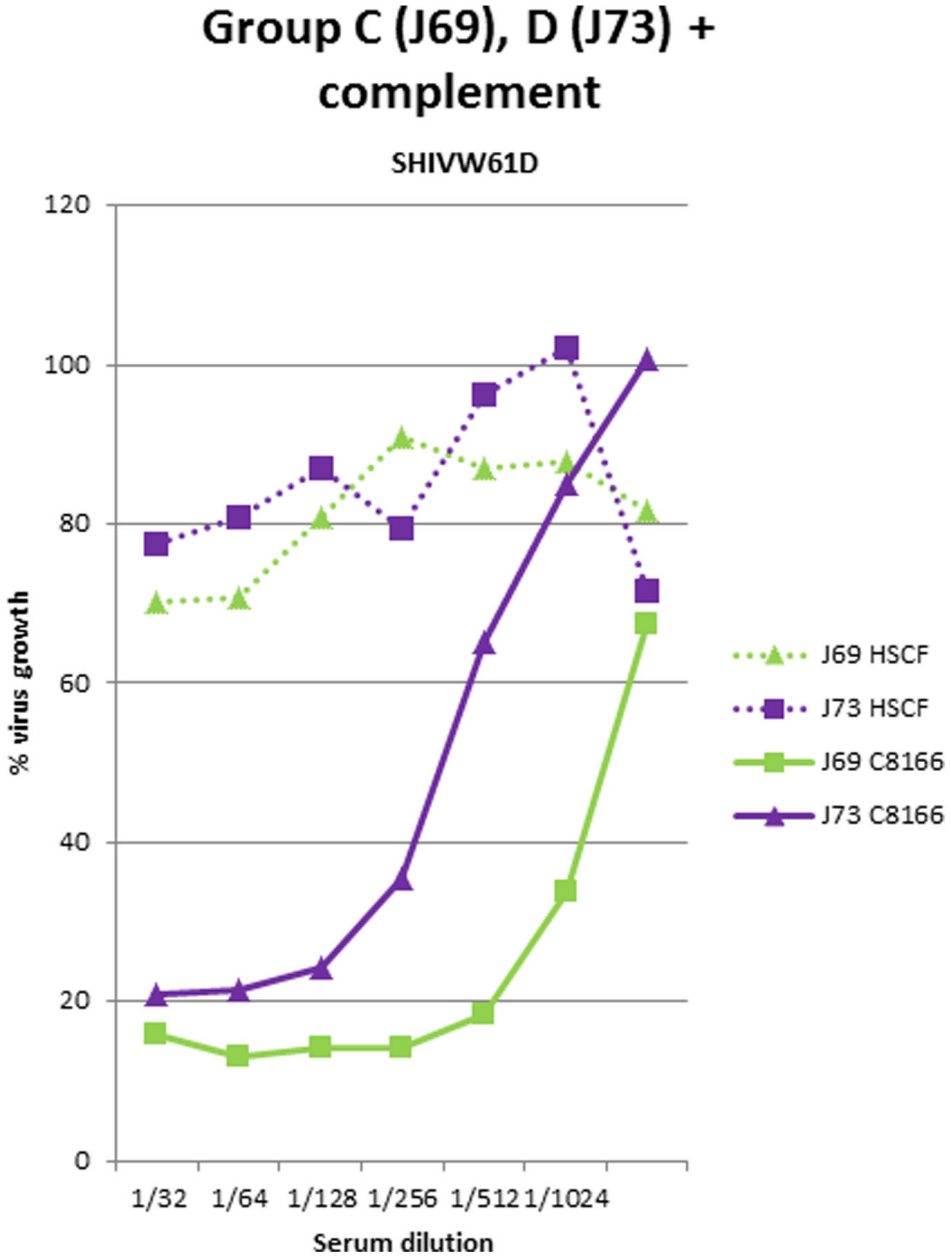
Infectivity neutralising activity against virus propagated on discordant cell substrate. Neutralising antibody activity against SHIV_W61D_ propagated on either human C8166 cells (solid lines) or macaque HSC-F cells (dotted lines) in sera from macaques immunised with SIV-infected (J69; green lines) or uninfected C8166 cell (J73; purple lines) vaccines.

In previously reported studies [40], protection was observed in immunised macaques only when they were challenged with SIV that had been propagated on the same human cell lines as those used to prepare the vaccines. By contrast, no protection was observed when macaques were challenged with viruses derived from macaque cells [20]. We sought to investigate whether the complement dependent neutralising activity detectable in the serum of macaques after immunisation with fixed inactivated vaccines was similarly dependent upon whether the virus had been propagated on human or macaques cells. SHIV_W61D_ stocks were prepared by passage of the virus stocks used in the assays described above on the macaque-derived cell line, HSC-F [37]. Heat inactivated sera collected from macaques immunised with either inactivated SIV (J69; group C) or uninfected C8166 cells (J73; group D) vaccines exhibited detectable neutralisation of SHIV_W61D_ propagated on the concordant C8166 cell, but no activity was observed when the same virus was propagated on the simian HSC-F cell line (Figure 7).

## Discussion

The development of vaccines that are able to generate potent anti-viral antibody responses against a broad range HIV-1 strains, would be a major breakthrough. Several years ago, it was demonstrated that inactivated viral vaccines prepared using a number of different approaches were able to protect macaques against detectable infection with SIV, even when challenged by the intravenous route [14], [15], [31]. Furthermore, this vaccine protection could be transferred with serum alone [14], indicating strongly that the vaccine protection was antibody mediated. However, in these studies vaccine efficacy was attributed to an immune response against cellular antigens (chiefly HLA proteins) incorporated into SIV during the budding process and which are common between both the vaccine and challenge virus [17]. The crucial evidence for this was that immunisation with uninfected cells identical with those used to propagate the challenge virus could also elicit a protective response [20]. However, in none of these preceding studies, was it elucidated why the potent protection conferred with anti-cell antibodies had not been recapitulated with anti- virus envelope protein antibodies. In this study, we sought to apply serological assays not available at the time of the original vaccine studies, to dissect the anti-viral properties of sera from macaques that had received inactivated vaccines and been challenged with virus. Analysis of sera from vaccinated macaques, within groups where only a proportion of vaccinates were protected, has identified assays able to establish correlations between specific serological responses and vaccine protection. These conclusions inform us of the properties of antibodies that an AIDS vaccine needs to elicit to deliver potent anti-HIV protection.

At the time when fixed inactivated vaccine studies were performed, it was hypothesised that a likely candidate responsible for inducing protective immunity in these macaque studies was human leucocyte antigens (HLA) as these comprise a significant proportion of the viral proteome [17]. Studies using HLA class I and II based vaccines were conducted [21], [22], [40] and protection was observed which supported this hypothesis. In this study, we have re-examined the sera from those original vaccine studies in more detail, using new methodologies. These new data identified that the magnitude of the anti-HLA antibody response elicited, by immunisation with fixed inactivated SIV and measured using a Luminex® based assay, discriminated between protected and infected macaques. The association between the magnitude of the response determined by the HLA bead-array assay and protection was most telling within vaccine groups where only a proportion of immunised individuals were protected. In all cases, both the anti-HLA Class I and anti-HLA Class II responses were lowest in those members of the group that became infected upon virus challenge.

By using a panel of Luminex® beads expressing different HLA allotypes, it was shown that the anti HLA responses in the immune sera was directed against framework determinants, since there was equal reactivity to multiple antigen bead sets. These data contrast with those we have reported previously, when we dissected the specificity of anti HLA class I and class II responses in HIV infected individuals administered a fixed inactivated HIV immuno-therapeutic “Remune”. In that report, responses in sera were directed against specific HLA alleles, suggesting that the responses were allotypic. Furthermore the responses following treatment with Remune did not break tolerance [29]. This distinction in the epitope specificity of anti HLA antibodies should be considered when extrapolating from xeno-immunisation studies in macaques to allo-immunisation in man and its potential as an AIDS vaccine.

The second assay that-was applied to analyse sera, was the neutralisation of virus infectivity using the TZM-bl cell line [33] as the target cell. Earlier neutralisation assays used permissive T cell lines such as C8166 and required assays to run for several weeks. As a result, the assays yielded confounding data arising from the cytotoxic effects of anti-HLA antibodies during long term cultures. The TZM-bl based assay measures virus infection (not virus replication), and so the assays are not protracted and cytoxicity is minimised. Using the infectivity assays, virus neutralisation was examined in the presence and absence of normal macaque serum, as a source of complement. In addition to the cells, different virus stocks were used to dissect the specificity of the virus neutralising activity. Stocks of both SIV and SIV/HIV-1 envelope chimera (SHIV_W61D_) [34] were used that had been prepared on either the human T cell line C8166 [36] or the simian T cell line HSC-F [37]. With these assays, we were able to discriminate between complement dependent and independent neutralisation targeted at viral and host cell proteins. Since both SIV and SHIV stocks were neutralised in a complement dependent manner when passaged on human T cells line and there are no reports of cross reactivity between HIV-1 and SIV envelope proteins, we conclude that vaccine protection correlated with complement mediated neutralisation of the virion by antibodies that were primarily specific for human host cell antigens. This was the case irrespective of whether inactivated virus, virus infected cells or inactivated uninfected cells were used as the vaccine. Thus, we conclude that this neutralising activity is directed against non SIV proteins such as the anti-HLA response detected with the Luminex® based assay.

The critical feature required to demonstrate a correlation between the virus neutralising antibody activity and protection against virus challenge was the titre of neutralising antibodies detected in the presence of complement. Although virus neutralisation was detectable in many serum samples in the absence of complement, the ability to differentiate protected from unprotected animals was not apparent until complement was added (Figures 4 and 5). Our data obtained in assays, performed without added complement, mirror those obtained by others who have been unable to detect a correlation between complement independent virus neutralisation and protection following immunisation with inactivated SIV vaccine, using a variety of assay formats [41]. Furthermore, others have reported previously that anti-cell antibodies, elicited by fixed inactivated SIV vaccines, are able to mediate virolysis in the presence of complement [21], [45]. Nevertheless, the data presented in this study are the first to demonstrate that the titre of these complement mediated neutralising antibodies correlate with the vaccine mediated protection against infection. This is due to our access to sera from vaccine studies where only a proportion of vaccinated individuals were protected and the application of a vaccine infectivity based virus neutralisation assay.

Whilst the titres of complement mediated neutralising antibody protection correlated with protection, they were not remarkably high. This is consistent with the reports of Spear et al. [42] and Montefiori et al [45], that reported virolysis was detectable in serum at dilutions between 1/30 and 1/300. Nevertheless even with these apparently moderate serological titres, potent reductions in virus titres were demonstrable *in vitro* [45]. This might suggest that only moderate titres of antibody are required when anti-viral antibodies are able to effectively engage complement. Previously it has been demonstrated that neutralising activity that engages complement induces virolysis [43]. Whether this is the only mechanism which applies *in vivo* is not known. The key advantage arising from the activation of complement is that it enables antibodies that are not of the highest affinity and in relatively low concentration to inactivate incoming virions. Theoretically, by engaging the complement system, one antibody molecule may lyse many virions.

If the correlation between vaccine mediated protection and the titre of anti-virion antibodies capable of engaging complement-mediated neutralisation proves indeed to be the mechanism, then there are a number of implications. No evidence of complement mediated neutralisation was detected in immune sera when they were tested against SIV propagated on the macaque cell line HSC-F. Similar data have been obtained previously by others [21], [45]. Thus, *in vivo* protection mediated in this manner must be extremely potent as it must be achieved by the neutralisation of every virion in the infectious inoculum, since all progeny virus will no longer express human cellular antigens. This is remarkable since sterilising immunity was reported by a number of groups [11], [12], [23]. At the same time, these data highlight a challenge for HIV vaccine design. Whilst fixed inactivated vaccines are able to elicit anti-cell antibodies that are able to engage complement to neutralise virus, the same vaccines appear completely unable to elicit anti-virus envelope antibodies with the same functional properties. The reasons for this are unclear. Although complement mediated antibody neutralisation has been described against HIV it has only been reported in sera from infected individuals [44]–[46] and not in vaccinated subjects. This may highlight important differences between the immunogenicity of natural virus infection and HIV-1 vaccines, which have yet to be explained. One possibility is that envelope spike density is too low and precludes bivalent binding by neutralising antibody [47]–[49] necessary for complement binding. A more intriguing possibility is that the remote topography of the neutralising antibody epitopes on the trimer spike or the high level of glycosylation is not compatible with efficient engagement and delivery of the complement membrane attack complex on to the virion membrane. The latter is supported by the report of Jiang et al [50].

Hessell at al [51] showed that engineering out the complement binding activity of an anti- HIV-1 envelope antibody did not impact on the protection observed when it was passively administered to macaques prior to SHIV challenge. This observation supports our view that anti-SIV gp140 envelope antibodies do not engage complement effectively and are unable to cause complement mediated virolysis. Thus the neutralisation activity of envelope specific antibodies function through receptor blockade mechanisms which requires both high titre and high affinity antibodies to achieve sterilising immunity *in vivo* [52]. By contrast, anti-host cell antibodies generated by fixed inactivated virus vaccines appear more readily able to engage the complement system. Particularly potent antibodies are those directed against HLA Class I and II antigens, whether they are generated in macaques by vaccination [13]–[15], [22] or derived from the serum of poly-transfused humans [44]. Attempts to recapitulate this sero-reactivity by allo-immunisation would appear more difficult [53]. Of course the specificity of anti-HLA antibodies generated by fixed inactivated vaccines in an allo situation are distinct [25] compared with the data presented in this paper (Fig. 1 and 2). In addition, antibody responses to further host cell antigens may contribute to the protection in a xeno-immunisation setting that would not be present in an allo-immune response. Of particular interest are responses to the complement regulatory proteins CD46, CD55 and CD59 [45]. The detection of these proteins on virions may account for the inability of complement to mediate virolysis, unless the blocking antibodies generated by xeno-immunisation are present. However, more recent data by Morner et al [54]have shown that macaques immunised with purified recombinant MHC Class I and II are also able to generate virus neutralising antibodies that exhibit enhanced titres when complement is present in the assay. Thus the anti-complement regulatory protein response is unlikely to be critical.

Engaging the complement system with anti-HIV antibodies needs to be undertaken with caution. Detailed analysis of infection sera from individuals has identified that prior to the appearance of serological responses that neutralise in vitro, non-neutralising antibodies can enhance virus uptake and infection of cells by engaging complement [55], [56]. This is of concern and a detailed understanding of the early antibodies that facilitate virus uptake in these *in vitro* assays is required. However, in a vaccine scenario, it is envisaged that the immune response will have matured further beyond this period of increased susceptibility before individuals encounter the virus.

The retrospective analysis of the protective serological responses generated by fixed inactivated virus vaccines informs the current interest in antibody mediated protection contributing to an effective AIDS vaccine. At the moment, most emphasis is focused on identifying how to generate high affinity, broadly cross reactive binding antibodies that block gp120/CD4 binding [10] and the analysis of required Ig V region sequences suggest that highly complex immunogens and immunisation regimens are likely to be required to elicit these responses [9]. We believe that these new data identifying an *in vitro* correlate of complement-mediated virus neutralisation with serum-mediated protection in the SIV/macaque model warrants further research into why engaging the complement system is capable of eliciting potent sterilising protection in the context of anti-cell responses and how this potent viral killing mechanism is so effectively defeated in the context of anti-HIV gp160 proteins. Overcoming this functional block to antibody mediated protection may prove to be an easier hurdle to overcome than the issue of antibody specificity.
